# Nitroglycerine

**Published:** 1895-10

**Authors:** 


Nitroglycerine, three drops a day of a 1 per cent. solution, is a powerful anti-neuralgic, especially in persistent sciatica.

